# Lack of Globulin Synthesis during Seed Development Alters Accumulation of Seed Storage Proteins in Rice

**DOI:** 10.3390/ijms160714717

**Published:** 2015-06-30

**Authors:** Hye-Jung Lee, Yeong-Min Jo, Jong-Yeol Lee, Sun-Hyung Lim, Young-Mi Kim

**Affiliations:** Department of Agricultural Biotechnology, National Academy of Agricultural Science, Rural Development Administration, Jeonju-si 560-500, Korea; E-Mails: leehyejung79@gmail.com (H.-J.L.); whdudalsv@naver.com (Y.-M.J.); jy0820@korea.kr (J.-Y.L.); limsh2@korea.kr (S.-H.L.)

**Keywords:** globulin deficiency, seed storage protein, 2-DE, seed storage organelle, expression analysis

## Abstract

The major seed storage proteins (SSPs) in rice seeds have been classified into three types, glutelins, prolamins, and globulin, and the proportion of each SSP varies. It has been shown in rice mutants that when either glutelins or prolamins are defective, the expression of another type of SSP is promoted to counterbalance the deficit. However, we observed reduced abundances of glutelins and prolamins in dry seeds of a globulin-deficient rice mutant (Glb-RNAi), which was generated with RNA interference (RNAi)-induced suppression of globulin expression. The expression of the prolamin and glutelin subfamily genes was reduced in the immature seeds of Glb-RNAi lines compared with those in wild type. A proteomic analysis of Glb-RNAi seeds showed that the reductions in glutelin and prolamin were conserved at the protein level. The decreased pattern in glutelin was also significant in the presence of a reductant, suggesting that the polymerization of the glutelin proteins via intramolecular disulfide bonds could be interrupted in Glb-RNAi seeds. We also observed aberrant and loosely packed structures in the storage organelles of Glb-RNAi seeds, which may be attributable to the reductions in SSPs. In this study, we evaluated the role of rice globulin in seed development, showing that a deficiency in globulin could comprehensively reduce the expression of other SSPs.

## 1. Introduction

Storage proteins constitute up to ~12% of the seed dry weight in cereal plants and these proteins are predominantly founded in the aleurone and subaleurone layers of the seed endosperm [1]. These seed storage proteins (SSPs) later become the amino acid and organic nitrogen reserves for embryonic and seedling development.

In rice (*Oryza sativa* L. japonica type) seeds, glutelins are clustered into types A, B, C, and D, according to a sequence homology analysis [2,3,4]. Most glutelin genes are expressed in the endosperm of rice grains. Promoter studies using the β-glucuronidase (*GUS*) reporter gene have shown that the promoters of several *GLUTELIN* genes (*GluA-1*, *GluA-2*, *GluA-3*, and *GluB-3*) are active in the peripheral region of the endosperm, whereas GUS expression driven by the promoters of *GluB-5*, *GluC*, and *GluD* was detected in various regions of the starch endosperm [4,5,6]. Time course profiles of seed protein abundances demonstrated that the abundance of glutelin proteins increases constantly in developing rice seeds from 7 to 23 days after flowering (DAF) [7].

Prolamins can be divided into three groups based on their electrophoretic mobility on sodium dodecyl sulfate (SDS)-polyacrylamide gels: 10, 13, and 16 kDa [8]. Proteins belonging to the 13-kDa prolamins are further subdivided into 13 kDa-I, 13 kDa-IIa, 13 kDa-IIb, and 13 kDa-III [9]. The prolamin genes are expressed chronologically during seed development, with the maximum levels of expression between 8 to 20 DAF [10], with the continuous accumulation of prolamin proteins to 21 DAF [11].

The major globulin in rice (α-globulin) contains 186 amino acids, forming the 21-kDa precursor protein [12]. Several research groups [13,14] have confirmed that the rice endosperm contains a single gene locus for globulin. Interestingly, the N-terminal nucleotide sequence of the globulin gene is very similar to those of high-molecular-weight (HMW) wheat glutenin (48% homology) and barley d-hordein (45% homology) [14]. The paralogous relationship between globulin and HMW glutenin suggests that the globulin gene is an essential progenitor of evolutionary events in higher plants [15]. The temporal expression of the globulin mRNA increases from five days after heading (DAH) and reaches its highest level at 30 DAH [16]. Its promoter is active in almost the entire rice endosperm [5], indicating the widespread expression of globulin in the rice endosperm during seed development.

A number of rice mutants have been reported that are defective in SSP synthesis and the posttranslational regulation of SSPs. The second type of mutant is mainly attributable to loss-of-function mutations in molecules associated with the proteolytic processing or intracellular transport of SSPs. These mutations were generated with chemically induced mutagenesis, T-DNA insertion, or RNA interference (RNAi) [17,18,19,20]. For instance, previous studies have noted that the seed endosperm of a mutant for endosperm storage protein 2 (*esp2*) displayed atypical accumulation of proglutelin compared with wild type, and an absence of molecular chaperone protein disulfide isomerase like 1-1 (PDIL1-1) [21,22]. The *esp2* mutant results from a mutation in the rice *PDIL1-1* gene, which functions in proglutelin maturation and probably in directing proglutelin and prolamin assembly on the endoplasmic reticulum (ER) cisternae. *Sar1s*-deficient mutants (*Sar1a/b/c*) and a mutant for glutelin precursor accumulation 3 (*gpa3*) display excess amounts of proglutelin, reductions in the glutelin acidic and basic subunits, and reduced globulin in the rice endosperm, leading to a floury seed phenotype [20,23]. *Sar1a/b/c* are genetically defective in the genes encoding GTPase members (*OsSar1a*, *b*, and *c*) of the COPII subunit, which is associated with the transport of proteins from ER to golgi apparatus. The *gpa3* mutant has a deficiency in the plant-specific kelch-repeat protein (GPA3), which regulates the trans-golgi network. Based on phenotypic analyses of these mutants, it is highly likely that the intracellular sorting pathway tightly controls the distribution and accumulation of SSPs during seed development.

The first type of mutants arises from the direct disruption of endogenous *SSP* gene expression [24,25,26]. Some rice mutants, such as *glu1*, *glu2*, and *glu3*, are characterized by the deletion of glutelin family spots from two-dimensional electrophoresis (2-DE) gels [27]. Molecular studies have shown that the *glu1* mutant is induced by the RNA-splicing-mediated silencing of the *GluB-5* gene, and is also designated “low glutelin content 1” (*lgc1*) [24]. A single mutation in the *GluB-5* gene resulted not only in a deficiency of the target protein, but also a reduction of glutelin A protein, because the sequence similarities between the glutelin species result in their co-suppression when double-stranded RNAs are formed [24]. Large-scale quantitative profiling of SSP expression was undertaken using rice knockdown (KD) mutants [25]. In single KD lines (*GluB*-less, 13-kD *Pro*-less, 10-kD *Pro*-less, and 16-kD *Pro*-less) and double KD lines (*Glu*-less and *GluB*·*Glb*-less), the expression levels of both targeted and nontargeted storage proteins were changed relative to those in wild type. For example, globulin and 13-kDa prolamin species were increased in the Glu-less line, where the expression of both GluA and GluB was inhibited. The expression of glutelins, globulins, and 10- and 13-kDa prolamins was relatively increased in the 16-kD Pro-less mutant, in which the expression of 16-kD prolamin species was repressed. It has been proposed that the supplementary expression of the *SSP* genes might compensate for the experimentally mediated deficiency of gene products in those KD mutants [25], although the compensatory mechanism responsible for inducing the expression of the endogenous *SSP* genes has not yet been identified. In addition to this unknown regulatory mechanism, some questions still remain. Are the different types of storage proteins functionally redundant, even when they are synthesized and stored in different places in the organelles, as they occur in rice seeds? Does this compensatory regulation take place invariably in all types of KD or knockout (KO) mutants related to *SSP* gene deficiency? Further work is needed to test whether there are consistent patterns in the compensation induction on SSP abundances.

Previous SSP studies have characterized the molecular localization of storage proteins and the formation of the storage organelles in rice seeds. These studies have investigated various aspects of cellular processes, including protein synthesis, intracellular transport mechanisms, and endosperm development. Studies of mutants with defects in seed-specific storage proteins have also suggested that there are different molecular requirements for the accumulation of SSPs under different conditions, altering the expression of storage protein gene families and the differentiation of storage organelles, generating different seed phenotypes. This report describes a transgenic rice plant, designated Glb-RNAi, in which endogenous globulin expression is inhibited in the seeds. We conducted various molecular analyses with immature and mature seeds obtained from Glb-RNAi transformants, and examined the expression profiles of SSPs and their related chaperone molecules. The results extend our understanding of the regulation of SSP accumulation and the comprehensive packaging of SSPs during seed development.

## 2. Results

### 2.1. Globulin Deficiency in Glb-RNAi Seeds

Transgenic rice plants carrying an RNAi cassette designed to target 299 nucleotides of the globulin transcript were initially identified with a herbicide-resistance test. Several independent transgenic lines were obtained, and two lines (Glb-RNAi 1–6 and 1–9) containing a single copy of the transgene were selected with a Southern blot analysis. The transformants were cultivated to at least the T_2_ generation, and most experiments described in this paper were performed with T_3_ seeds.

**Figure 1:**
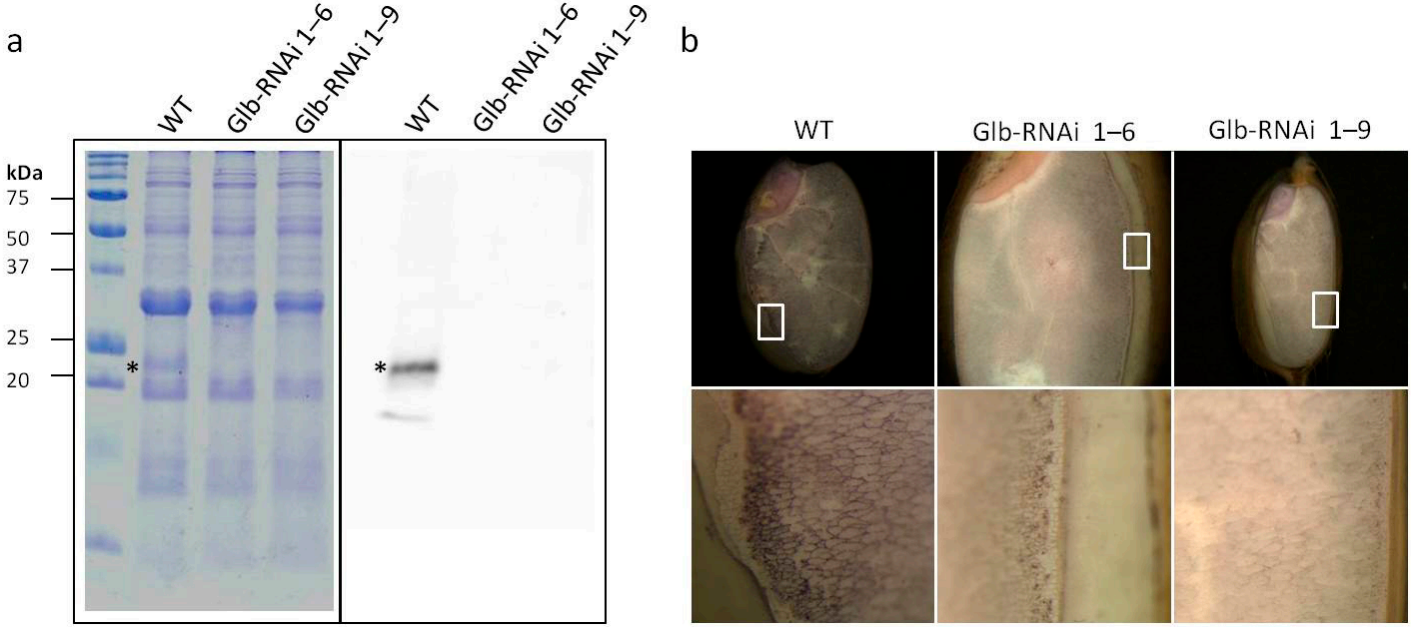
Expression analysis of the globulin gene product in RNAi-mediated transformant seed. (**a**) Western blotting analysis of the total proteins isolated from a single rice grain from wild type (WT) and transgenic lines (Glb-RNAi 1–6 and 1–9). The asterisk indicates the protein recognized by the anti-globulin antibody; (**b**) *In situ* western hybridization was performed to detect the globulin protein in intact seeds. Sections of mature seeds obtained from WT and transgenic lines (Glb-RNAi 1–6 and 1–9) were treated with anti-globulin antibody and visualized under a microscope. The **upper** panels show images of the entire section of the stained seed, and the areas marked with a white box are magnified in the **lower** panels.

To confirm the expression of endogenous globulin in the Glb-RNAi transformants, the total proteins from dry seeds were separated and visualized with an SDS-PAGE analysis. A particular band that was similar in size to the rice 21-kDa globulin was present in wild-type extracts but absent from the extracts of both Glb-RNAi lines (Figure 1a, left panel). Moreover, the signal recognized by an anti-globulin antibody was clearly observed in wild-type protein extract but was undetectable in the protein extracts from the Glb-RNAi plants (Figure 1a, right panel).

Intact seeds were also examined for the presence of globulin protein. A longitudinal section of a rice grain was incubated with the globulin-specific antibody and observed under a light microscope. As shown in Figure 1b, the color development induced by the antibody was apparent on the sections of wild-type seeds. The seeds were densely stained in the thin outer layers of the endosperm, indicating tightly bound antibody in the aleurone and subaleurone layers. In contrast, the staining corresponding to the same antibody reaction was either pale or invisible on the sections of Glb-RNAi seeds. These data indicate that the final product of the target gene was dramatically decreased in the dry seeds of the RNAi-mediated transgenic rice plants.

### 2.2. Analysis of Storage Protein Gene Expression Levels in Developing Seeds

**Figure 2:**
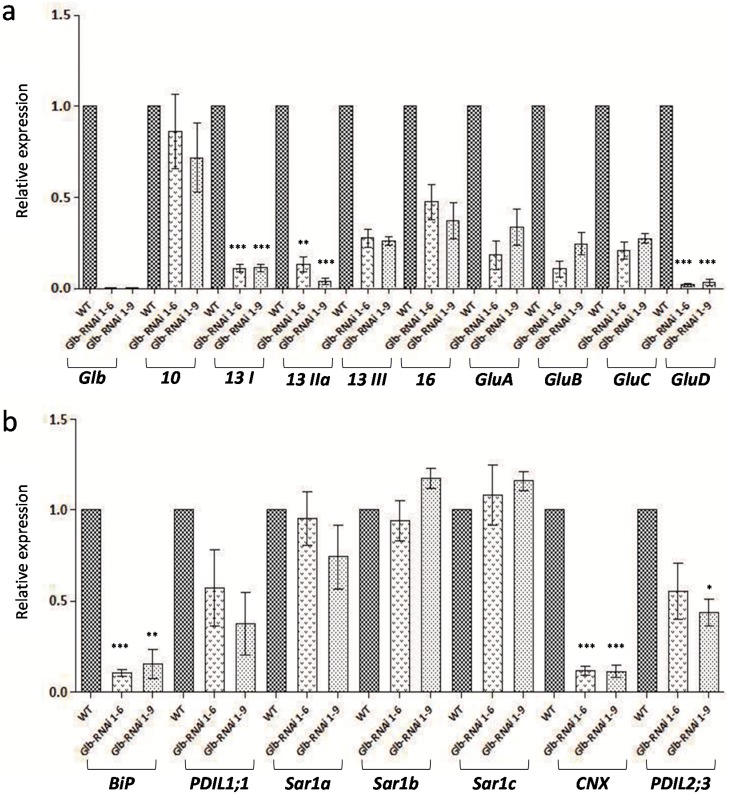
Quantitative real-time reverse transcription (RT)-PCR analysis of Glb-RNAi transformants. (**a**) Transcription levels of *SSP* genes; and (**b**) genes related to endoplasmic reticulum (ER)-localized chaperone proteins were analyzed in the immature seeds of wild type (WT) and Glb-RNAi 1–6 and 1–9 lines. Data are the means ± SD of three biological replicates. Statistically significant differences between the WT and RNAi lines are indicated by asterisks (* *p* < 0.05; ** *p* < 0.01; *** *p* < 0.001; *t*-test). Ubiquitin was used as the internal control gene in this analysis. *Glb*, globulin; *10*, prolamin 10 kDa; *13 I*, prolamin 13 kDa-I; *13 IIa*, prolamin 13 kDa-IIa; *13 III*, prolamin 13 kDa-III; *16*, prolamin 16 kDa; *GluA*, glutelin A; *GluB*, glutelin B; *GluC*, glutelin C; *GluD*, glutelin D; *BiP*, binding protein; *PDIL*, protein disulfide isomerase like; *Sar1 a*–*c*, Ras related GTPase 1 homologues a, b and c; and *CNX*, calnexin. The primers used for RT-PCRs are informed in Table S1.

To observe the relative expressions of various *SSP* transcripts in the Glb-RNAi lines, their seeds were harvested at two weeks after flowering, when the rice grains had entered the ripening phase and were filled with starch, protein, lipid, and secondary metabolites. The absence of globulin mRNA was demonstrated with qPCR (Figure 2a).

The expression of three genes belonging to the 13-kDa prolamin group was markedly reduced in the transformant seeds relative to their expression in wild-type seeds. In particular, the expression of the genes encoding 13 kDa-I and 13 kDa-IIa was downregulated about 0.1-fold (*p* < 0.001). The expression of the 16-kDa-type prolamin gene was about 0.5-fold less in Glb-RNAi seeds than that in wild-type seeds, whereas the expression of the 10-kDa-type prolamin gene was comparatively unaffected (Figure 2a). The transcriptional expression of the glutelin genes was also examined in cDNAs obtained from the immature Glb-RNAi seeds. To define the abundances of particular glutelin transcripts encoded by the multigene family, four genes (*GluA-1*, *GluB-1a*, *GluC-1*, and *GluD-1*), one from each glutelin group, were tested [4]. The mRNA levels of *GluA-1*, *GluB-1a*, and *GluC-1* were approximately 0.7- to 0.8-fold less than wild-type levels in the transformant seeds. *GluD-1* showed the lowest level of all the glutelin genes in both Glb-RNAi lines (*p* < 0.0001 and *p* < 0.0003). Thus, the transcript abundances of the several *SSP* genes studied showed lower levels in Glb-RNAi lines than in wild type.

The mRNA levels of genes related to the posttranslational processing of SSPs or genes encoding chaperone proteins were examined to comprehensively investigate the absence of globulin during seed development. The expression of *BiP*, which encodes a protein involved in the folding and accumulation of prolamin polypeptides, was reduced to around 80% of wild-type level in Glb-RNAi seeds. *PDIL1;1* is known to play an essential role in the oxidative folding of proglutelin and the proper distribution of seed proteins [21,28], and this *PDIL1;1* displayed up to 0.6-fold less in Glb-RNAi lines than that of wild type. The expression of the gene encoding the ER-specific chaperone calnexin (CNX) was also downregulated in the immature seeds of Glb-RNAi lines. The expression of *CNX* is reported to be regulated by ER stress [29,30]. Interestingly, the expression of *PDIL2;3*, which encodes a disulfide isomerase on the surfaces of protein bodies I (PB-Is) and is involved in the development of PB-Is by maintaining the polymerization of the prolamin proteins, was also limited in Glb-RNAi lines [31,32]. The transcription of genes encoding several SSP-related chaperones was obviously reduced in the developing seeds of Glb-RNAi lines (Figure 2b) in this study.

In contrast, the abundances of transcripts associated with protein secretion from ER to golgi (*Sar1a*, *Sar1b*, and *Sar1c*) were either maintained or slightly increased in the transgenic lines compared with their levels in wild type (Figure 2b). The Sar1 proteins, members of a family of small GTPases, exist in the ER compartment and function in the transportation of protein storage vesicle (PSV)-targeted proteins. Their transcription was almost unaltered in the immature seeds of Glb-RNAi lines. This pattern differs from that of the genes mentioned above, such as *BiP*, *CNX*, and the *PDIL*s.

### 2.3. Analysis of Protein Accumulation in Mature Seeds

It was observed that the levels of *SSP* transcripts were predominantly reduced in Glb-RNAi lines during seed development (Figure 2), the expression patterns of SSPs were also examined in dry seeds. The total seed proteins were isolated from both wild type and transformants, and separated into three fractions containing prolamin, globulin, and glutelin, according to their differential solvent solubilities: the glutelins in rice seeds are soluble in alkali solution [2], the prolamins are soluble in alcohol [33], and the globulin proteins are soluble in saline solution [34].

**Figure 3:**
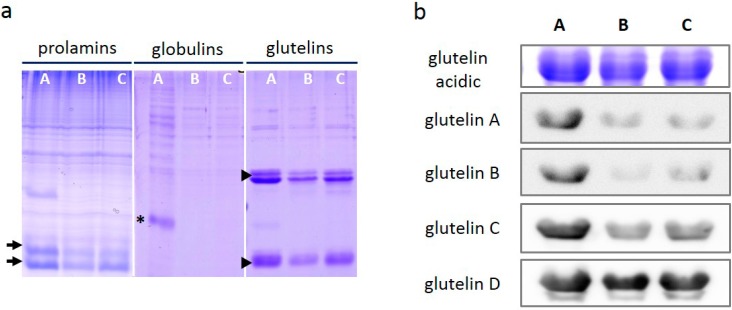
Accumulation of three different types of storage proteins in Glb-RNAi seeds. (**a**) Ground seed powder (50 mg) was fractionated based on solvent solubility, and the three fractionated extracts were resolved on an sodium dodecyl sulfate-polyacrylamide gel and stained in Coomasie Brilliant Blue (CBB) staining solution. arrows, prolamins; asterisk, globulins; arrow heads, glutelins; (**b**) The glutelin-containing extracts were used for an immunoblotting analysis. The immune signals recognized by the anti-glutelin antibodies were detected by a luminescent image analyzer (LAS-4000, Fujifilm). A, wild type; B, Glb-RNAi 1–6; C, Glb-RNAi 1–9.

The globulin protein band was only detected in the saline fraction from wild type, and was absent from the extracts from Glb-RNAi lines. A protein band similar in size to the globulin protein band was slightly detectable in the alcohol-soluble fraction of wild type, but not in the fractions from the transformants. Prolamins appeared in all the alcohol-soluble fractions, but the intensity of the bands was slightly lower in Glb-RNAi lines than in wild type (Figure 3a). Because most of the prolamin genes analyzed in this study showed restricted expression at the mRNA level in Glb-RNAi lines (Figure 2a), it eventually caused the accumulation of prolamins in the mature seeds (Figure 3a). Glutelins, which make up more than 50% of the total SSPs, were clearly visible in the alkali-soluble fraction from wild type. However, the accumulation of glutelins was substantially reduced in both transformant lines. The reduced transcript levels of glutelins in Glb-RNAi lines could lead to decreased accumulation of glutelin proteins in the mature seeds of the transformants.

An immunoblotting analysis confirmed again the reduced glutelin proteins in the mature seeds of the transformants. The band that was immunologically recognized by the anti-glutelin antibody was fainter in the protein extracts from both RNAi lines than in the protein extract from wild type (Figure 3b). A significantly reduced signal was observed in Glb-RNAi lines with antibodies directed against glutelin A, glutelin B, and glutelin C. However, the anti-glutelin D antibody signals were identical in wild type and Glb-RNAi seeds.

### 2.4. Comparative Solubility of Glutelins from Mature Seeds

Solubilization buffer containing a reducing agent was generally used in this study to isolate the storage proteins from dry and compact seeds. SSPs are reported to be highly aggregated through disulfide bonding [21], thus a reducing agent was deemed necessary in the buffer to separate the proteins from the seeds. A comparative analysis of the protein extraction was performed with and without the reductant (β-ME). The seed proteins were initially isolated from 100 mg of seed powder using a buffer without the reductant, and the remaining pellet was isolated in the presence of β-ME.

**Figure 4:**
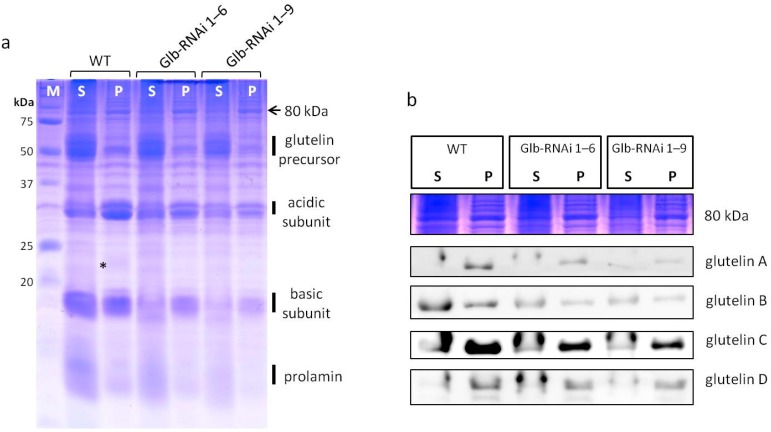
Extraction patterns of storage proteins from wild type (WT) and Glb-RNAi lines. (**a**) Proteins isolated in the absence (S) or presence (P) of β-ME were resolved on an SDS-PAGE gel and stained with CBB solution. M, unstained protein standard marker (Bio-Rad, #161-0363); *, globulins; (**b**) Proteins were transferred to polyvinylidene difluoride (PVDF) membranes and antibodies directed against various glutelins were used to detect the accumulated glutelin acidic proteins. CBB-stained protein band at approximately 80 kDa indicates the internal loading control. S, supernatant; P, pellet.

Globulin was predominantly seen in the pellet fraction from wild-type seeds, and the glutelin precursors and prolamins were predominantly found in the supernatant fraction (Figure 4a). Glutelin proteins were detected in both the supernatant and pellet fractions: one-third of the glutelin acidic subunits and half the basic subunits were found in the supernatant fraction, and the rest were detected in the pellet fraction (Figure 4a). Interestingly, glutelins displayed different CBB-staining patterns in wild type and Glb-RNAi seeds. The staining of the glutelin acidic subunit was significantly reduced in the pellet fraction from Glb-RNAi seeds compared with that from wild-type seeds (Figure 4a), although its expression was relatively stable in the supernatant fraction. The glutelin basic subunit was significantly reduced in the supernatant fractions from Glb-RNAi seeds relative to that from wild-type seeds, and the staining of the glutelin basic subunit was also reduced in the pellet fraction of Glb-RNAi seeds (Figure 4a). The expression of glutelins isolated with or without a reductant was also determined with an immunoblotting analysis using anti-glutelin antibodies. The signal intensities for the anti-glutelin A, anti-glutelin B, and anti-glutelin C antibodies in the pelleted fractions were lower in Glb-RNAi seeds than in wild-type seeds (Figure 4b). However, the anti-glutelin D antibody signals were almost identical in the pellet fractions of wild type and Glb-RNAi seeds. This expression pattern of glutelin D is identical to the result shown in Figure 3b. The expression and elution patterns of the glutelins were significantly altered in the mature seeds of the transformants, and the reduced levels of glutelin seemed to be caused by the disturbance of particular glutelin species.

### 2.5. Protein Profiles in Globulin-Deficient Seeds

Because the abundances of SSPs were generally reduced in the mature seeds of Glb-RNAi lines, we used a proteomic study to investigate which proteins were primarily affected by the lack of globulin. The total proteins were isolated from equal weights of seed powder from wild type and Glb-RNAi seeds in the presence of β-ME. The protein extracts were precipitated, and the entire proteins were subjected to IEF and SDS-PAGE analyses, as described in the Experimental Section.

**Figure 5:**
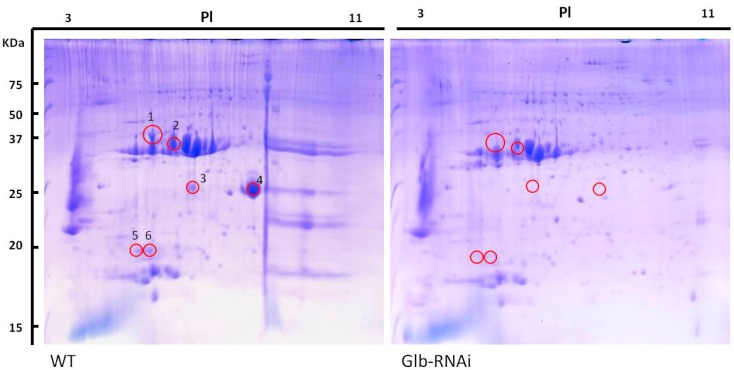
Isoelectric focusing and SDS-PAGE analyses of wild type (WT) and Glb-RNAi seeds. Equal amounts (20 mg) of seed powder from WT and Glb-RNAi transformant seeds were resolved in SDS-urea buffer and the total proteins were separated with 2-DE, as described in the Methods. The intensities of the labeled protein spots (shown inside the red circles) were lower in the protein extract from the transformants compared with those from WT extract. The CBB-stained 2-D gels were photographed and the images were analyzed with ImageJ. PI, isoelectric point.

After the 2-DE gels were stained, the gels were compared. Abundant spots were visible in wild-type gel, but a number of protein spots were only faint or were absent in the gel containing Glb-RNAi seed proteins (Figure 5). Six spots with clearly reduced intensities in the Glb-RNAi gels were excised for peptide sequencing. The predominant sequences derived from each protein spot were matched to peptides available in the National Center for Biotechnology Information (NCBI) database, with varying degrees of certainty. The fully matched proteins determined with a BLAST search are described in Table 1.

**Table 1:** Seed protein abundances that differed significantly between wild type and Glb-RNAi lines. Protein spots marked in Figure 5 were excised from the 2-D gels and the proteins were identified with LC–MS/MS and a BLAST search (only 100% matched proteins are included).

Spot #	Identified Protein	Coverage ^a^	Theor. *M*w (kDa) ^b^	Theor. Pi ^c^	Locus No.	Function
1	Glutelin type-B1	29.35	54.1	9.22	Os02g0249800	Storage protein
	Glutelin, provisional	21.14	53.5	9.03	Os02g0249600	Storage protein
	Hypothetical protein; similar to glutelin type-B2 precursor	17.82	54.0	9.22	Os02g0248800	Storage protein
	Glutelin type-B5	24.04	35.6	7.06	Os02g0268300	Storage protein
	Hypothetical protein; similar to aspartic proteinase oryzasin 1 precursor	7.66	55.8	7.05	Os01g0663400	Proteinase
	Blast and wounding induced mitogen-activated protein kinase	5.22	55.2	7.94	Os06g0708000	Kinase
2	Hypothetical protein; glutelin provisional	40.38	35.6	7.06	Os02g0268100	Storage protein
	Glutelin type-B1	18.03	54.1	9.22	Os02g0249800	Storage protein
	Glutelin, provisional	10.53	51.5	8.56	Os10g0400200	Storage protein
	Glutelin, provisional	9.25	51.2	8.68	Os01g0762500	Storage protein
	Transposon protein, putative	3.57	96.8	6.35	Os03g0380600	Plant transposase
	Hypothetical protein; bHLH-MYC and R2R3-MYB transcription factors	8.91	37.5	6.98	Os06g0233800	Transcription factor
3	Glutelin, provisional	11.01	51.2	8.68	Os01g0762500	Storage protein
	Cupin family protein	6.55	61.4	7.53	Os03g0336100	Cupin family protein
	Globulin	4.60	19.8	6.96	Os05g0499100	Storage protein
	Lysosomal thiol reductase family protein	8.20	28.6	6.24	Os03g0295800	Reductase enzyme
	Hypothetical protein; universal stress protein-like	14.12	18.2	6.71	Os03g0750000	Putative stress-related protein
	Hypothetical protein; similar to FAD-dependent oxidoreductase family	4.59	61.4	6.89	Os08g0114300	Reductase enzyme
4	Globulin	9.20	19.8	6.96	Os05g0499100	Storage protein
	Hypothetical protein; manganese-superoxide dismutase precursor	16.88	25.0	7.31	Os05g0323900	Superoxide dismutase family protein
	Cupin family protein	3.89	61.4	7.53	Os03g0336100	Cupin family protein
	Glutelin, provisional	5.51	51.2	8.68	Os01g0762500	Storage protein
	Pyruvate phosphate dikinase, provisional	2.74	88.1	5.63	Os05g0405000	Kinase
	Hypothetical protein; similar to germin-like protein precursor	13.26	19.6	8.72	Os08g0189100	Cupin family protein
5	Alpha-amylase inhibitors, provisional	16.25	17.3	8.03	Os07g0215500	Allergenic protein
	Hypothetical protein; similar to prolamin	42.38	17.0	8.72	Os07g0219400	Storage protein
	Alpha-amylase inhibitors, provisional	10.19	17.0	8.03	Os07g0214600	Allergenic protein
	Hypothetical protein; similar to early nodulin-like protein	6.74	19.3	8.34	Os08g0273300	Nodulin-like family protein
6	Alpha-amylase inhibitors, provisional	13.31	17.3	8.03	Os07g0215500	Allergenic protein
	Putative prolamin	8.15	17.0	9.17	Os05g0329200	Storage protein
	Prolamin	5.31	17.1	9.38	Os07g0219400	Storage protein
	Alpha-amylase inhibitors, provisional	3.81	17.0	8.03	Os07g0214600	Allergenic protein

**^#^** Number of the proteins spots in Figure 5; ^a^ Coverage means the percentage of the protein sequence covered by the identified peptides; ^b^ theoretical molecular weight (*M*w); ^c^ theoretical isoelectric point (pI).

Diverse proteins were identified, including SSPs, kinases, allergens, stress-related proteins, transcription factors, and hypothetical proteins, and over 31% and 10% of the identified proteins represented glutelins and prolamins, respectively. Despite our limited understanding of the particular properties and cellular functions of individual proteins, the proteomic data imply that the accumulation of storage proteins in dry seeds clearly differs in wild type and Glb-RNAi seeds. Our analysis confirms that a number of functional SSPs are largely absent in Glb-RNAi seeds.

### 2.6. Observation of Storage Protein Organelles in Immature Seeds Compared with Mature Seeds

**Figure 6:**
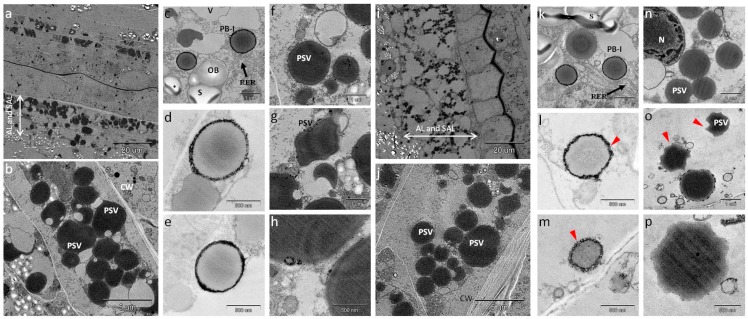
Phenotypic characterization of seed storage organelles. TEM was used to observe the protein bodies in developing seeds of wild type (**a**–**h**) and Glb-RNAi transformants (**i**–**p**). Scale bar is shown in each panel. AL, aleurone layer; SAL, subaleurone layer; CW, cell wall; V, vacuole; S, starch grain; OB, oil body; N, nucleus; PB-I, protein body I; PSV, protein storage vacuole; and RER, rough endoplasmic reticulum. Red arrowheads, distinguished outline and shape compared with those of wild type.

To clarify the formation of storage protein organelles in globulin-deficient rice, seeds were harvested from wild type and Glb-RNAi transformant plants in the early stage of grain ripening. Because the aleurone and subaleurone cells surrounding the starch endosperm provide a major reserve for the synthesis of PB-Is and PSVs in rice seeds, samples of the outer layers of immature seeds were prepared and analyzed with TEM (Figure 6a,i). The PB-Is were observed as spherical electrolucent bodies and the PSVs as irregular, highly electron-dense structures.

When a section was randomly selected for higher magnification, irregularly shaped and larger PSVs were observed more often than spherical and smaller PB-Is (Figure 6b,j), which is similar to the distribution ratio of storage organelles described by Krishnan and White (1995) [35]. The ER-derived PB-Is consisted of lightly stained concentric structures with an electron-dense ring, and were preserved well in the developing seeds of wild type and Glb-RNAi lines (Figure 6c,k). However, compared with the PB-Is observed in wild type (Figure 6d,e), the PB-Is in Glb-RNAi seeds frequently displayed an uneven or dull outline (Figure 6l,m; red arrowhead). In addition to PSVs with an asymmetric, highly electron-opaque structure (shown in Figure 6f–h,n), a certain number of PSVs in the transformants displayed a loosely packed and untidy structure (Figure 6o,p; red arrowhead). Furthermore, these PSVs were smaller than those in wild type. These observations indicate that the intracellular structures of the developing endosperm cells were hindered in Glb-RNAi transformants.

A phenotypic evaluation of seed development in the endosperm of Glb-RNAi T_3_ generation was performed. The mature seeds of Glb-RNAi and wild type were examined for shape and morphological patterns in the endosperm, including the aleurone cells and starchy endosperm (Figure S1). The aleurone cells in wild type contained cellular materials or content and had a rigid rectangular structure, which were also found in the transformants. The crystalline shape of the starch granules was observed in both wild type and Glb-RNAi lines. Similar quantities of total starch were also measured in wild type and Glb-RNAi grains (Figure S2). The rice grains of the Glb-RNAi transformants showed the same average shape and growth as wild-type grains (Figure S3). We detected no obvious differences in the developmental phenotypes of the mature seeds of the Glb-RNAi transformant and wild type.

## 3. Discussion

The International Rice Genome Sequencing Project and the International Rice Annotation Project Database (RAP Database) have shown that the rice globulin gene occurs at only a single locus on the 5th chromosome, differing in copy number from the other SSP genes, including those encoding prolamin and glutelin [15]. The rice genome contains at least 34 prolamin genes [10] and 15 glutelin genes [36]. The globulin protein is also known to constitute the smallest proportion of the total SSPs in rice grains. This is probably because the globulin is a single copy gene that no rice mutant in which only the expression of the globulin gene is modulated has been reported until now. However, *in vitro* and *in vivo* studies of the synthesis and targets of globulin in the rice endosperm have been reported, in which the globulin gene was either tagged with a fluorescent molecule or modified with a point mutation [31,37]. The globulin mRNA is detected in PB-Is, whereas the globulin protein is found in PSVs in the mature seeds. This is different from other SSPs, such as prolamin and glutelin. The entire process of prolamin synthesis occurs in the PB-Is, whereas glutelin mRNAs and polypeptides are observed in the ER and are then sorted into the PSVs. When the localization of globulin mRNA was disrupted, the sorting of glutelin was particularly disturbed in the ER.

Here, we have reported rice mutants with impaired globulin synthesis, which we generated with an RNAi-based strategy. We then analyzed the progeny seeds of the T_2_ generation of homozygous plants. We first observed that the mRNA expression of a number of SSPs was repressed in the developing seeds of the Glb-RNAi transformants (Figure 2a). The SSP profiles obtained with immunoblotting and 2-DE analysis of the dry seeds resembled their SSP transcript levels in most cases (Figure 3 and Figure 5). Specially, the altered patterns of glutelin proteins in the transformant seeds showed an identical result, even when single seeds were randomly selected from wild type and Glb-RNAi lines (Figure S4a). We confirmed that these patterns were preserved well between the generations of Glb-RNAi lines (Figure S4b). These results imply the coordinated accumulation of certain types of storage proteins together with globulin proteins when the rice endosperm is ripening.

Washida *et al.* (2012) [37] showed that the mis-sorting of globulin mRNA to the ER cisternae, instead of into the ER-derived PB-I, resulted in spatially aberrant patterns of both globulin and glutelin proteins in the rice endosperm. They suggested that a globulin–glutelin heteromeric complex may be formed for effective storage. The same research group also used density gradient fractionation to analyze the seed protein extracts from transgenic plants expressing globulin in either PB-Is or ER cisternae. Their SDS-PAGE analysis showed no differences between the protein abundance patterns for globulin and glutelin in either transformant. Thus, it seems that the modification of globulin localization did not affect or barely affected the accumulation and storage of glutelin. However, our data showed a reduction in glutelin synthesis in the absence of globulin. Because the glutelin proteins are known to polymerize with globulin in the PSVs, it is likely that a deficiency of globulin protein may affect the quantity and stability of other storage proteins during seed development. Several studies have shown that the mRNA levels of SSPs are increased in rice mutants in which the synthesis of one or several SSPs is reduced [24,25]. Nevertheless, in our study, the transcription of numerous SSPs was downregulated in Glb-RNAi lines, and the SSPs accumulated less in Glb-RNAi seeds than in wild-type seeds. In contrast to most SSPs, whose protein expression reflected their transcription levels, glutelin D showed different patterns between mRNA and protein expressions. The expression of the glutelin D gene was repressed at the transcript level, but the final product was typically induced and accumulated in the dry seeds of Glb-RNAi lines. These patterns suggest that complex regulatory mechanism underlie expression of individual SSPs or that a temporary recovery mechanism exists that induces SSP expression for the functional compensation of defective proteins.

In our study, the expression of, not only the SSP genes, but also some chaperone proteins associated with the posttranslation modification of SSPs were altered at the transcript level in the transformants. It has been previously reported in glutelin- and prolamin-deficient mutants that the SSP suppression induced regulation of ER-localized chaperones, such as BiP and PDI [25]. Our data show that the expression of these genes, including *BiP*, *PDIL1-1*, *CNX*, and *PDIL2-3*, was reduced in Glb-RNAi lines, where a parallel universal reduction of SSPs was observed. The *OsSar1* genes were unchanged compared with the other genes tested. The small GTPase Sar1 is involved in the COPII complex, which functions in vesicular trafficking on membranes, and the *Sar1a/b/c*-RNAi mutant showed abnormal patterns of PSV-targeted storage proteins in the seed endosperm [20]. However, the deficiency of globulin and the reduction in glutelin did not affect the transcriptional regulation of the *Sar1* genes in Glb-RNAi lines. In our study, BiP, PDI, and CNX, which are potentially involved in the folding and stability of SSPs, were influenced in Glb-RNAi lines, but this was not observed for *Sar1*, which is involved in the SSP-sorting pathway, implying that the regulation of the intracellular mechanisms related to storage protein accumulation in the rice endosperm are complex and specific. Considering the universal downregulation of SSP expression at both the transcription and translation levels, it is noteworthy that globulin may play a critical role in a transcriptional mechanism and in the *de novo* protein maturation process of storage proteins in the rice endosperm.

## 4. Experimental Section

### 4.1. Plasmid Preparation

The pANDA-β plasmid construction was as described previously [26]. A 299-base-pair (bp) fragment of rice globulin cDNA was amplified with PCR using the pANDA-Glb primers (shown in Table S1). The fragment was inserted into both sides of the *gus* region in the antisense and sense orientation through *att*R recombination cassette, driven by a maize ubiquitin 1 promoter.

### 4.2. Plant Transformation

The constructed binary plasmids were introduced into rice calli induced from the Korean rice cultivar *Ilmi* (japonica-type). Transformation was according to the method of Kim *et al.* (2012) [38].

### 4.3. In Situ Western Hybridization

*In situ* western hybridization was performed as described previously [39]. Briefly, mature seeds were partitioned into two lengthwise sections. The seed sections were washed in distilled H_2_O, and then soaked in Tris-HCl-SDS (125 mM Tris-buffered saline (TBS), pH 6.8, and 2% (*w*/*v*) SDS) for 30 min. The sections were preincubated in blocking solution (TBS (pH 7.5), 3% (*w*/*v*) skimmed milk) for 3 h, and then incubated overnight at room temperature in blocking solution containing the appropriate primary antibody (TBS, 1% (*w*/*v*) skimmed milk, and primary antibody diluted 1:250). The sections were washed three times with TBS. A goat anti-rabbit IgG antibody conjugated with alkaline phosphatase (AP; Promega, Madison, WI, USA) was used as the secondary antibody. The ProtoBlot^®^ II AP System with Stabilized Substrate (Promega) was directly applied to detect signals from the antibody. All images were recorded with the Leica M205C Microsystem (Leica, Heerbrugg, Switzerland).

### 4.4. Sodium Dodecyl Sulfate (SDS)-Polyacrylamide Gel Electrophoresis (PAGE) and Immunoblotting

The total seed proteins were extracted from one grain of rice in 350 μL of SDS-urea buffer (4% SDS, 8 M urea, 0.25 M Tris-HCl (pH 6.8), 20% glycerol, 0.01% bromophenol blue, and 5% β-mercaptoethanol (β-ME)). The supernatant was removed after centrifugation at 10,000× *g* for 10 min. The proteins were separated on 15% SDS-PAGE gels. For protein staining, the gels were soaked in Coomassie Brilliant Blue (CBB) staining solution (0.1% (*w*/*v*) CBB R-250, 45% (*v*/*v*) methanol and 45% (*v*/*v*) glacial acetic acid) for 3 h, and washed in destaining solution (1% methanol, 1% glacial acetic acid, and 8% distilled H_2_O). The gels were blotted onto polyvinylidene difluoride membranes (PVDF; Perkin Elmer, Boston, MA, USA) for immunoblotting. The membranes were blocked at room temperature for 2 h in blocking solution (TBS and 5% skimmed milk) containing the appropriate primary antibody. A horseradish-peroxidase (HRP)-conjugated anti-rabbit IgG antibody (Promega) was used to detect the globulin- and glutelin-responsive antibodies and an AP-conjugated anti-rat IgG antibody (Promega) was used to detect the prolamin-responsive antibody. A luminescent image analyzer (LAS-4000, Fujifilm, Tokyo, Japan) was used to visualize signals from the antibodies on the membranes.

### 4.5. RNA Extraction and Gene Expression Analysis

Immature rice seeds were frozen in liquid nitrogen and stored at −80 °C until use. Total RNA was extracted from the seeds with a plant RNA purification reagent (Plant RNA Reagent) (Invitrogen, Carlsbad, CA, USA). To eliminate genomic DNA contamination, the RNA was treated with DNaseI (TURBO™ DNase; Ambion, Carlsbad, CA, USA) and purified again with the RNeasy Plant Mini Kit (Qiagen, Hilden, Germany). The RNA (1 μg) was used to synthesize complementary DNA (cDNA) in the presence of oligo(dT)_20_ primers using the SuperScript III First-Strand Synthesis System (Invitrogen), according to the manufacturer’s instructions. The cDNA was made up to 100 μL with water, and its concentration was measured with a NanoDrop 1000 spectrophotometer (Thermo Scientific, Wilmington, DE, USA). An aliquot (10 ng) of diluted cDNA was then used for quantitative PCR (qPCR), performed with AccuPower^®^ GreenStar qPCR Master Mix (Bioneer, Daejeon, Korea) and the appropriate primer pairs on a C1000 thermal cycler with the CFX96^®^ Real-Time Detection System (Bio-Rad, Foster City, CA, USA). The cycling parameters were as follows: 15 min at 95 °C, 40 cycles of 10 s at 95 °C, 10 s at 55 °C, and 30 s at 72 °C, and then a gradient from 65 to 95 °C to produce the melting curve. The primer sequences used for the qPCR are shown in Table S1. Each data value and standard deviation (SD) presented in this manuscript is the mean of three biological replicates, and the value for each sample was obtained from the mean of three replicated PCR reactions.

### 4.6. Seed Storage Proteins (SSP) Fractionation

Rice grains were finely ground with a mortar and pestle. The rice flour (100 mg) was rinsed with 400 μL of acetone at 4 °C for 30 min. After centrifugation at 10,000× *g* for 10 min, the pellet was dissolved in phosphate-buffered saline (PBS; 0.866 M K_2_HPO_4_, 0.134 M KH_2_PO_4_ (pH 7.6), and 0.4 M NaCl). The sample was incubated at 4 °C for 1 h, and the supernatant containing globulin was isolated. The flour was then extracted in 55% *n*-propanol and centrifuged to obtain prolamin. After the flour was washed with distilled H_2_O, glutelin was extracted with 1% lactic acid containing 1 mM ethylenediaminetetraacetic acid (EDTA). A 10 μL aliquot of each SSP fraction was processed for SDS-PAGE analysis.

### 4.7. Two-Dimensional Gel Electrophoresis (2-DE)

The total seed proteins were isolated from 20 mg of fine rice flour made from either wild type or Glb-RNAi seeds in SDS–urea buffer. The proteins were precipitated overnight in 15% trichloroacetic acid (TCA), followed by dissolving in 0.35 mL of 32.5 mM CHAPS rehydration buffer containing 18.2 mM dithiothreitol (DTT). The entire protein suspension was subjected to both isoelectric focusing (IEF) using Immobiline DryStrip pH 3–11 NL (GE HealthCare, Uppsala, Sweden), according to the manufacturer’s instructions, and SDS-PAGE. Three replicates from three independent protein extracts were tested. In-gel digestion and a mass spectrometric analysis of the spots of interest were performed by the National Instrumentation Center for Environmental Management (Seoul National University, Seoul, Korea).

### 4.8. Microscopic Analysis

Transmission electron microscopy (TEM) was used to analyze the immature seeds from both wild type and Glb-RNAi plants grown in a GMO field (N 37°15′48.8617″ E 127°1′42.9632″). The seeds were fixed overnight in 1.25% glutaraldehyde and 2% paraformaldehyde in 50 mM PBS at 4 °C. After the specimens were washed in PBS, they were dehydrated in a graded series of ethanol and embedded in Epon 812. The specimens were sliced into ultrathin sections with an ultramicrotome (Leica). The sections were stained with a solution of uranyl acetate and lead citrate and observed with TEM (LEO912AB, Carl Zeiss, Jena, Germany).

## Supplementary Materials

Supplementary materials can be found at http://www.mdpi.com/1422-0067/16/07/14717/s1.
