# Supplementary material for: Lack of Globulin Synthesis during Seed Development Alters Accumulation of Seed Storage Proteins in Rice
Source: Int J Mol Sci. 2015 Jun 30;16(7):14717–36. doi: 10.3390/ijms160714717 (PMC4519868; doi:10.3390/ijms160714717)
Supplement: Supplementary file 1 [file ijms-16-14717-s001.pdf]

Supplementary Information

Table S1. Primer list used in this study.

Target Gene	Primer Name	Forward Primer	Reverse Primer	GenBank
Globulin	*pANDA-Glb*	AAAAAGCAGGCTGAGAGGTTCCAGCCGA	AGAAAGCTGGGTCTCGCCCTGGTCAGC	GQ848069
	Glb	AGTCGGAGATGAGGTTCAGG	GAACATCGGCTGGAACCTC	
Prolamin 10 kDa	*10*	TTATTTGTGCTGGACTCGGG	GAGAGTTGGAAGTTGACAGGG	EF122448
Prolamin 13 kDa-I	*13 I*	CAACTACAGTCGCATCTCCTAC	GGGTTGCCACTATGCTATACTG	EF122447
Prolamin 13 kDa-IIa	*13 IIa*	GCTCTGTTGGCTTTTAACGTG	ACTCATTACAAGACACCGCC	GU120358
Prolamin 13 kDa-III	*13 III*	TCACCCGTGTTTCAACTGAG	CACAATAGCCTGAACACTGC	FJ940200
Prolamin 16 kDa	*16*	CTCAATTTGCCCTCCATGTG	AGAACCGCAATGACCAGTAG	EF122449
Glutelin A	*GluA*	AATGATGGTGAAGTGCCGGT	TCACGCCTGTATGCTTGAGG	EF122456
Glutelin B	*GluB*	ATTGAGCAACACTCTGGGCA	TGGCTCTGTAGCCTCTTTGC	EF122460
Glutelin C	*GluC*	CACAAGGGCCAATAGCCAGA	GGTCACGTACATCACCGTGT	EF122465
Glutelin D	*GluD*	AAGACAGAGCGACCAAGCTC	ATGTGCAACACTAGCCGGAA	EF122464
Binding protein	*BiP*	AGGACATCAGCAAGGACAAC	GGACTCAATCTCAACACGGAC	AK065743
Protein disulfide isomerase like 1;1	*PDIL1;1*	AACGATGTGCCAAGCGAGTTCGAT	TTAGAGCTCATCCTTGAGAGGCTC	AB373950
GTP-binding protein Sar1a	*Sar1a*	AGTGTTGTCCGCAAGATGGG	CGCTGGGCAGAGTATGCAAG	AK112012
GTP-binding protein Sar1b	*Sar1b*	GCAAGATGGGCTATGGGGA	TGGTAAGGTGAAACAGGAGTATGAAC	AK099149
GTP-binding protein Sar1c	*Sar1c*	GCGTCGTCCGCAAGATG	AGGAGAGTTGATAAACAGAACCAGAG	AK119548
Calnexin	*CNX*	TCGACAACCCCAACTACAAAG	ATCTCAATCCCAATAGCGGC	AK069118
Protein disulfide isomerase like 2;3	*PDIL2;3*	ATAAGAGGATTTCCAACTATTAAG	TGCTCCTTGATAATCTACTG	AP005559

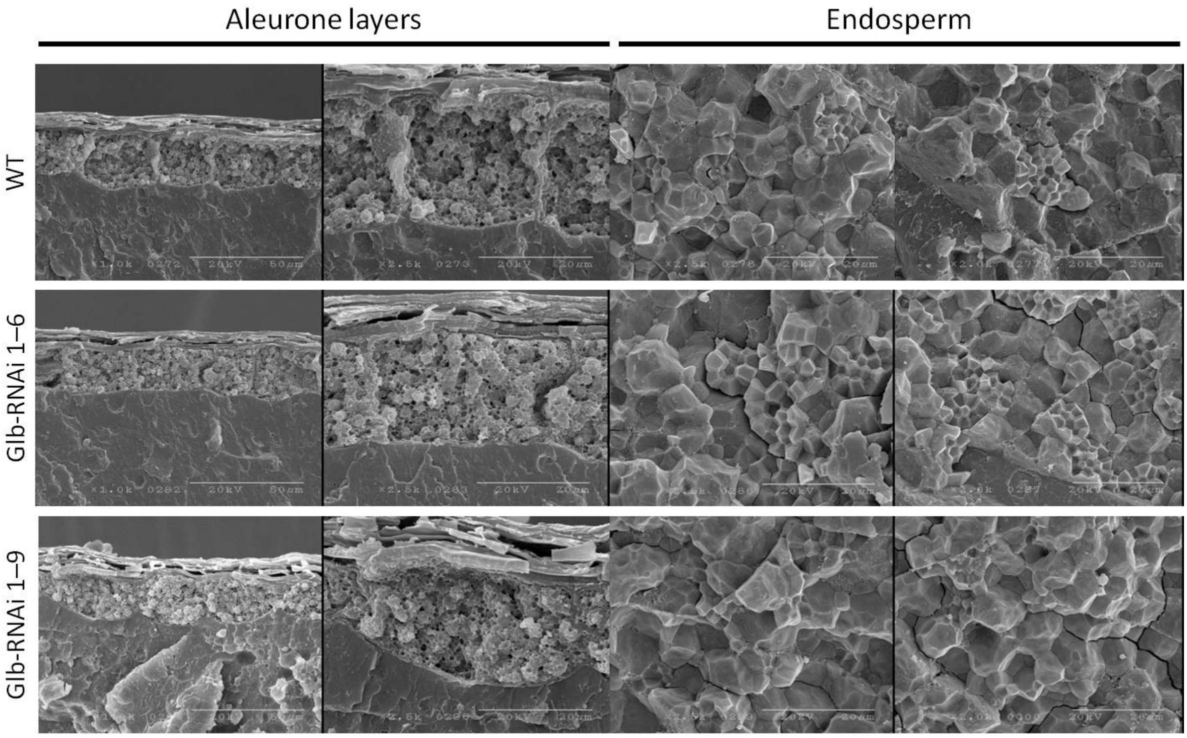

Figure S1. Phenotype analysis of seed endosperm by scanning electron microscopy. Seeds were transversely sectioned using a sharp knife and prepared on a specimen slice. Aleurone layers as well as starch granules were observed in wild type (WT) and Glb-RNAi lines. Comparative analysis of endosperm morphology indicates no significant differences between wild type and transformants.

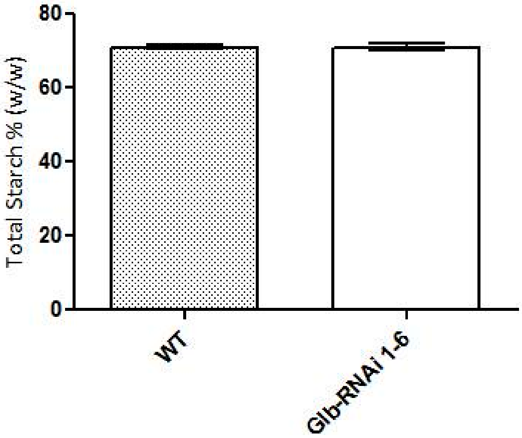

Figure S2. Measurement of starch content in wild type (WT) and Glb-RNAi line. An equal amount (100 mg) of seed powder was prepared from WT and Glb-RNAi 1–6. Total starch measurement was performed using TOTAL STARCH Kit, as described by the manufacturer (Megazyme). This analysis indicates that starch content was similar between the wild type (71.059 ± 0.944) and Glb-RNAi 1–6 lines (71.004 ± 1.959).

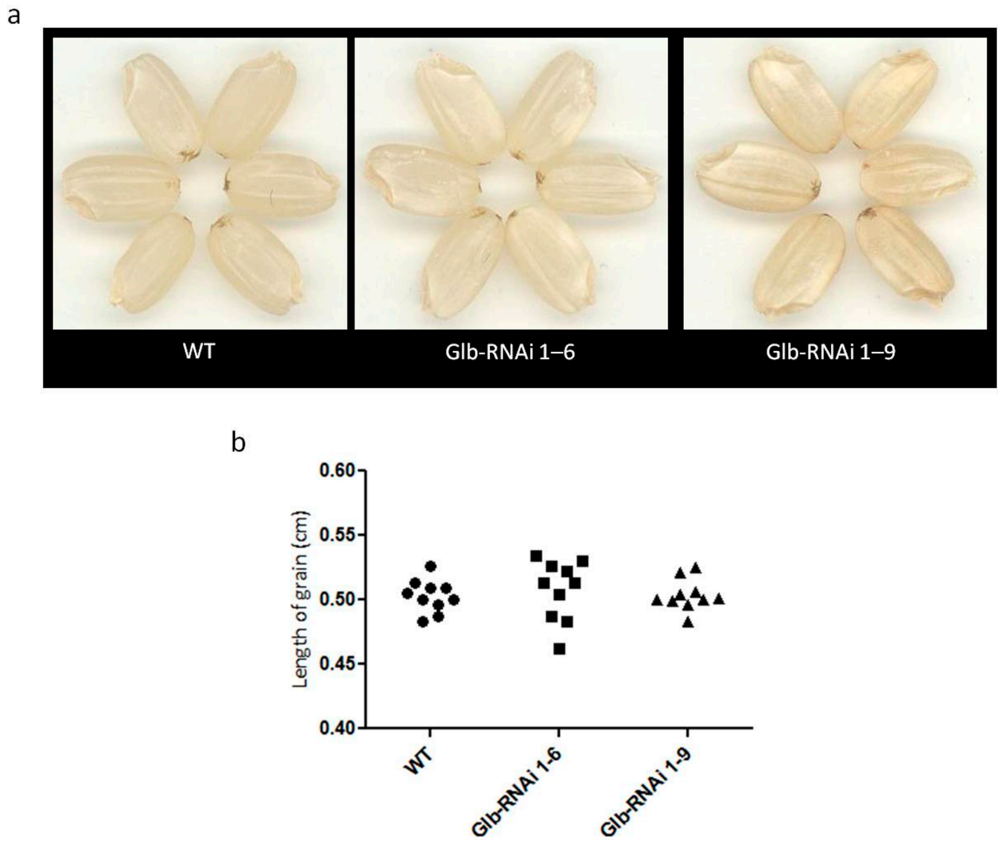

Figure S3. Phenotypic analysis of seed growth. (**a**) The photos indicate comparative grain sizes for the wild type (WT) and Glb-RNAi transformants; (**b**) Ten seeds were randomly selected from the WT and Glb-RNAi lines, and grain size was measured using ImageJ.

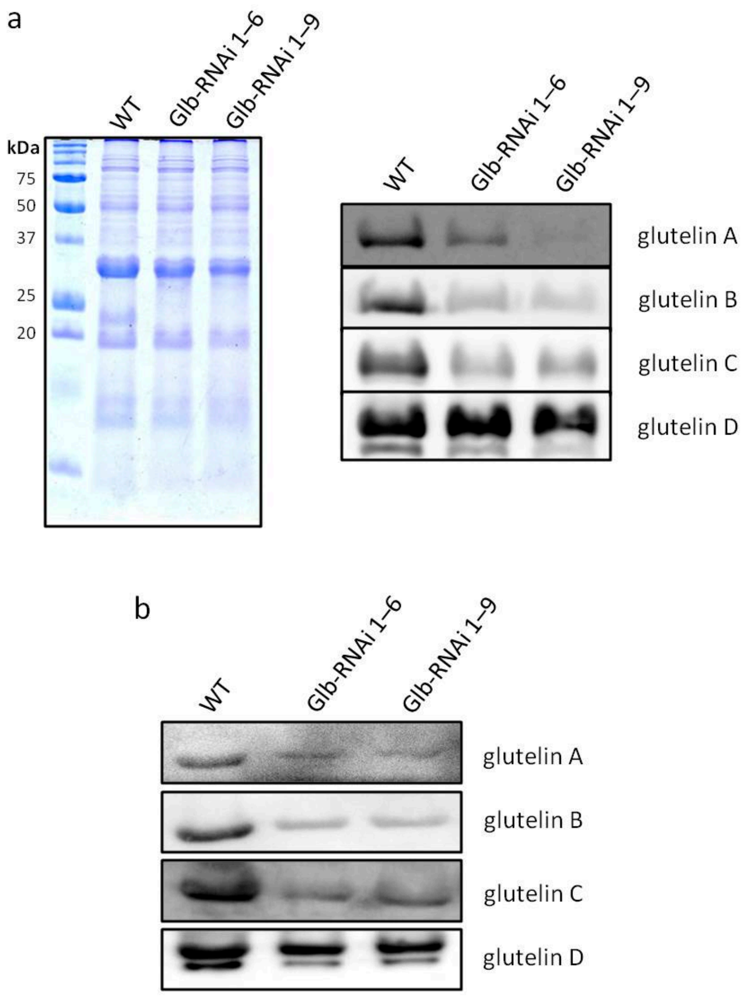

Figure S4. Immunoblotting analyses using anti-glutelin antibodies. (**a**) Total protein was extracted from one seed which was selected at random from both wild type (WT) and Glb-RNAi lines. Proteins were loaded onto SDS-PAGE and further transferred onto PVDF membranes. These membranes were incubated with diverse anti-glutelin antibodies. The signal was detected and recorded by a luminescent image analyzer (LAS-4000, Fujifilm); (**b**) Proteins used in this analysis were isolated from dry seeds of T_1 generation of WT and Glb-RNAi transformants. The experiment process was identical to the method described previously.
